# Aberrant anatomy angel – a near-miss penetrating neck trauma and a life-saving retropharyngeal right subclavian artery

**DOI:** 10.1259/bjrcr.20220104

**Published:** 2023-09-11

**Authors:** Sanjana Vijayan Menon, Kreyen Ponen, Aman Berry Williams

**Affiliations:** 1 Bond University, Robina, Australia; 2 The University of Queensland, Brisbane, Australia; 3 The University of New England, Armidale, Australia

## Abstract

Penetrating neck injuries constitute a relatively rare subset of trauma, which unfortunately carries with it significant morbidity and mortality. In the Emergency Department (ED), rapid clinical decompensation related to haemorrhagic, obstructive or mixed damage to major vessels and airways is typically the primary culprit, which is compounded even further by any intrathoracic involvement. Even rarer, however, is to sustain such an injury with no haemodynamic compromise and follow through with an uneventful clinical course. Here we present a remarkable case of a dirt-bike accident which left a male impaled by a tree branch, and the swift clinical conduct along with the fortuitous variation in his anatomy that saved his life.

## Case presentation

A 40-year-old gentleman was brought in by ambulance to the ED of a major tertiary hospital. Several hours earlier, he had been riding a dirt-bike on the beach when he veered into a neighbouring shrub, fell off the vehicle and impaled his right neck on a large tree branch. With the tree branch still embedded, he was speaking in full sentences and able to provide a detailed history on arrival to the ED. He denied loss of consciousness, shortness of breath or any overt worsening neck or chest pain. All vital signs were well within normal limits. He did, however, have reduced apical breath sounds in the left chest. On careful examination of the tree branch, it entered the right neck base, travelling in a lateral-to-medial direction towards the thoracic inlet. There was no evidence of active bleeding, and no expansile or pulsatile haematoma. He had a full complement of upper limb pulses, intact cranial nerve function on neurological examination, as well as grossly unremarkable upper and lower limb function.

A chest X-ray was performed in the ED resuscitation bay, revealing a left-sided pneumothorax which did not warrant immediate insertion of a chest drain. By this time, the ED team was joined by Anaesthetics, members of the Intensive Care Unit, as well as Vascular, Ear Nose and Throat, and Cardiothoracic surgical subspecialties. Given his stability and the absence of worrying signs such as airway compromise, stridor or massive haemorrhage, the consensus was made to obtain a computed tomographic angiography (CTA) to assess for further neck and intrathoracic injury, and to plan a surgical approach dependant on the involved structures with emergency operating theatres on standby.

## Imaging findings

**Figure 1.**
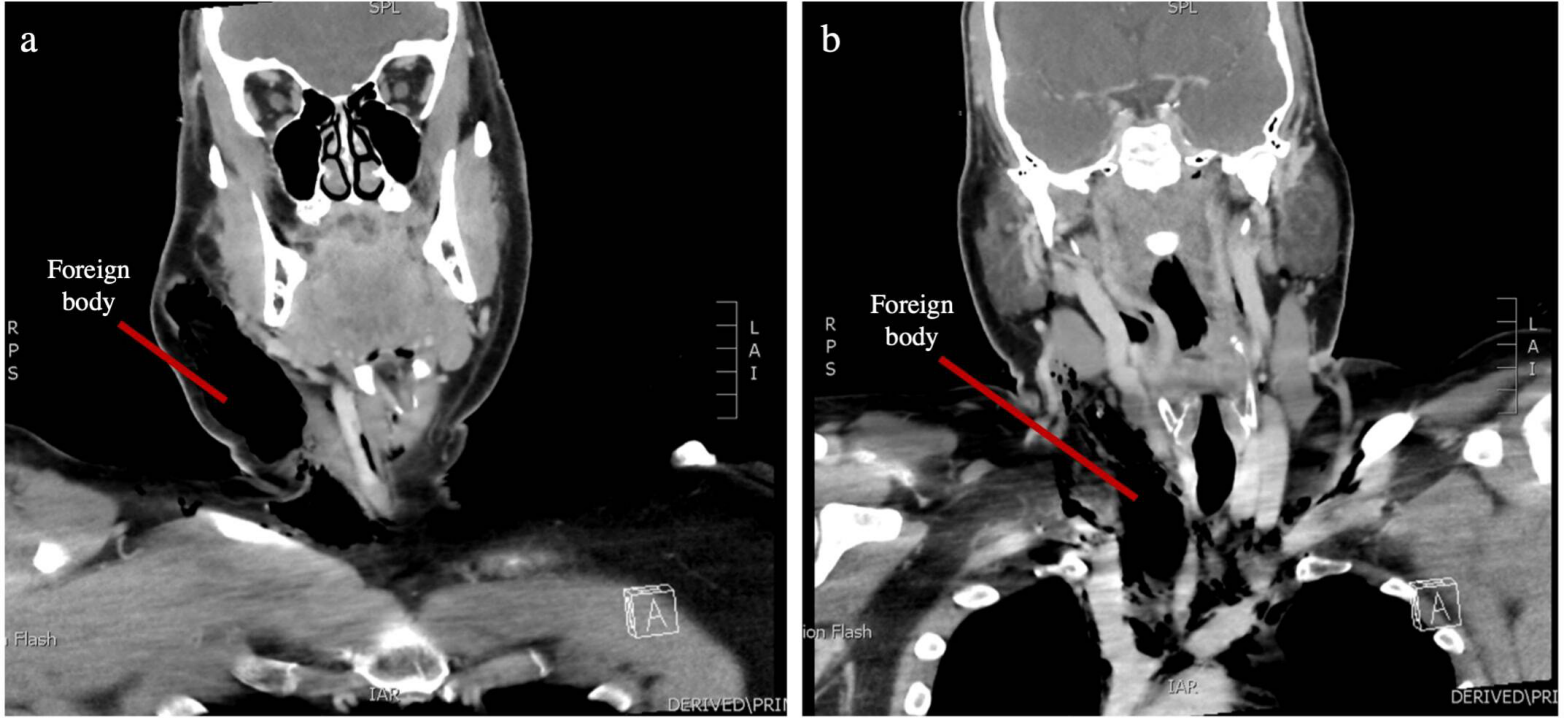
(**a and b**) Coronal CTA scans showing the hypodense wooden foreign body in the right neck.

**Figure 2.**
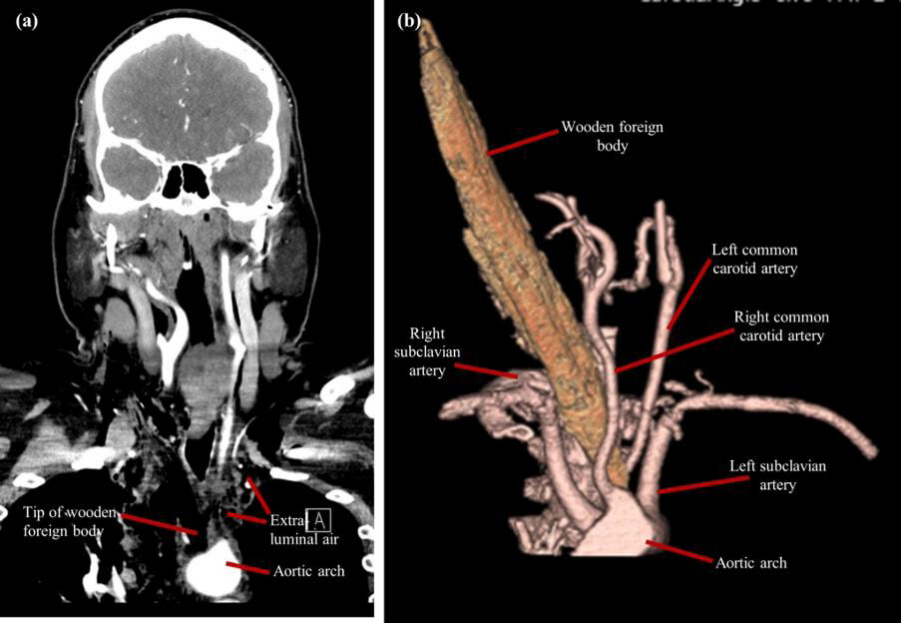
(**a and b**) An anterolateral CTA with 3D volume rendering showing the presence of the wooden foreign body amongst the major vessels originating from the aortic arch, with an apparent absence of the brachiocephalic trunk. The aberrant right subclavian artery is visualised travelling retropharyngeal, posterior to the foreign body.

**Figure 3.**
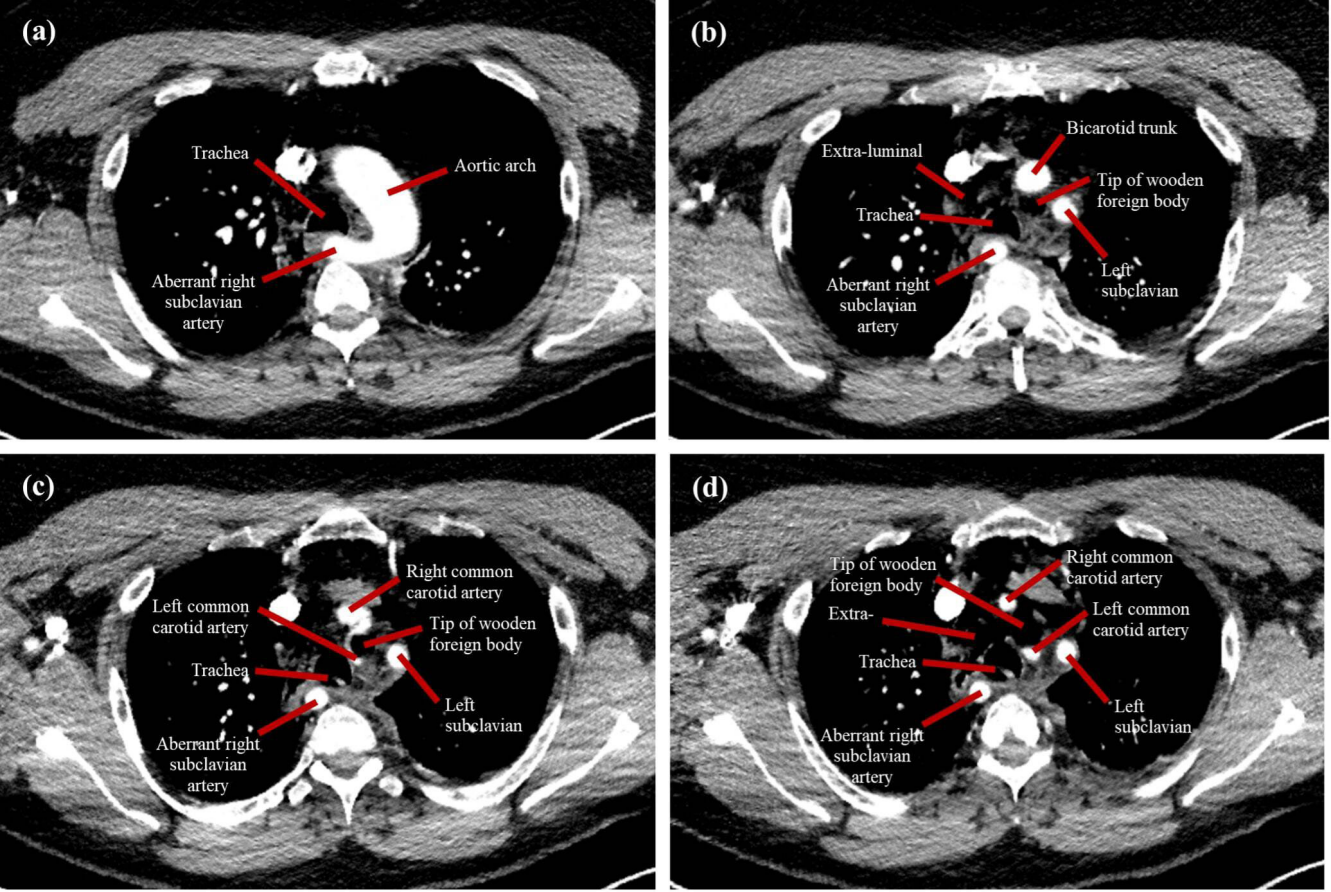
(**a, b, c and d**) Gallery of axial CTA images showing the pathway of the wooden foreign body through the major vessels of the aortic arch. Note the presence of the bicarotid trunk giving rise to the left and right common carotid arteries, as well as the retro-oesophageal course of the aberrant right subclavian artery. The left common carotid artery is visualised coursing around the medial aspect of the foreign body.

The information yielded from the CTA was quite surprising. The foreign body was clearly visualised (Figure 1), with its tip between the origin of the left common carotid artery and left subclavian artery, closely abutting the medial portion of the aortic arch (Figure 2). There was noted to be an aberrance of the origin of the right subclavian artery, arising directly from the aortic arch and travelling retropharyngeal, which sustained no injury (Figure 3). The right common carotid artery was medially displaced, and the right internal jugular vein was anteriorly displaced, with suspected compression; however, there was no apparent traumatic pseudoaneurysms or active extravasation of the major vessels. Although there was no insult to the airway, the patient had a mildly displaced and compressed trachea, most likely as a result of the position of the foreign body. CTA also confirmed the left-sided pneumothorax and showed pneumomediastinum, seen as extraluminal air (Figure 3). These injuries were likely due to the penetrating foreign body, considering there was breach of the pretracheal fascia, thereby introducing air into this space. CT abdomen was unremarkable, with no intraperitoneal free fluid present (not shown).

## Treatment

Capitalising on his haemodynamic stability, he was taken to theatre for a neck dissection and hemi-sternotomy to remove the foreign body, which did not require intraoperative cardio-pulmonary bypass. Microscopy, culture and sensitivity samples were sent prior to washing out the area, with safe removal of the wooden foreign body. With a mediastinal and neck drain in place, he was admitted to the intensive care unit and intubated, whilst on Piperacillin/Tazobactam and inotropes. Following extubation a day later, he reported minimal pain at the sternotomy site; however, he had a mild seroma but with no signs of infection. A video fluoroscopic swallowing exam also showed no obvious contrast leak outside the pharyngeal or oesophageal lumen, with no apparent mechanical obstructions or distortions of anatomy.

## Follow-up

He was reviewed by the Cardiothoracic Team following discharge from the hospital, who noted well-healed wounds and a stable sternum, with a referral for physiotherapy to improve range of motion of his neck. He returned a year later, complaining of sternal click exacerbated by coughing and upon movement of shoulders, with no evidence of a ventral wall hernia on ultrasound. He also experienced intermittent dysphagia and gagging on eating, often resulting in an episode of explosive coughing, which was reviewed by the Vascular team; however, this symptom was not of significant concern for him. He subsequently had a sternal fixation done with plates, due to non-union at the level of mid-sternum. The patient was extremely pleased with the procedure and was discharged from the hospital after regular follow-up.

## Discussion

Penetrating neck and chest trauma pose a significant morbidity and mortality risk due to the vascular structures in the anatomical region. Hence, immediate advanced life-support measures and prompt multidisciplinary input is vital to a successful outcome in the management of these patients. Often complex in its mechanism of injury, these cases can be further complicated by risks posed by the foreign body itself, such as sepsis, embolism, vascular injuries and pneumothorax.^1^ 25% of cases are complicated by vascular trauma, with mortality rates reported to be as high as 50%.^1^

**Figure 4.**
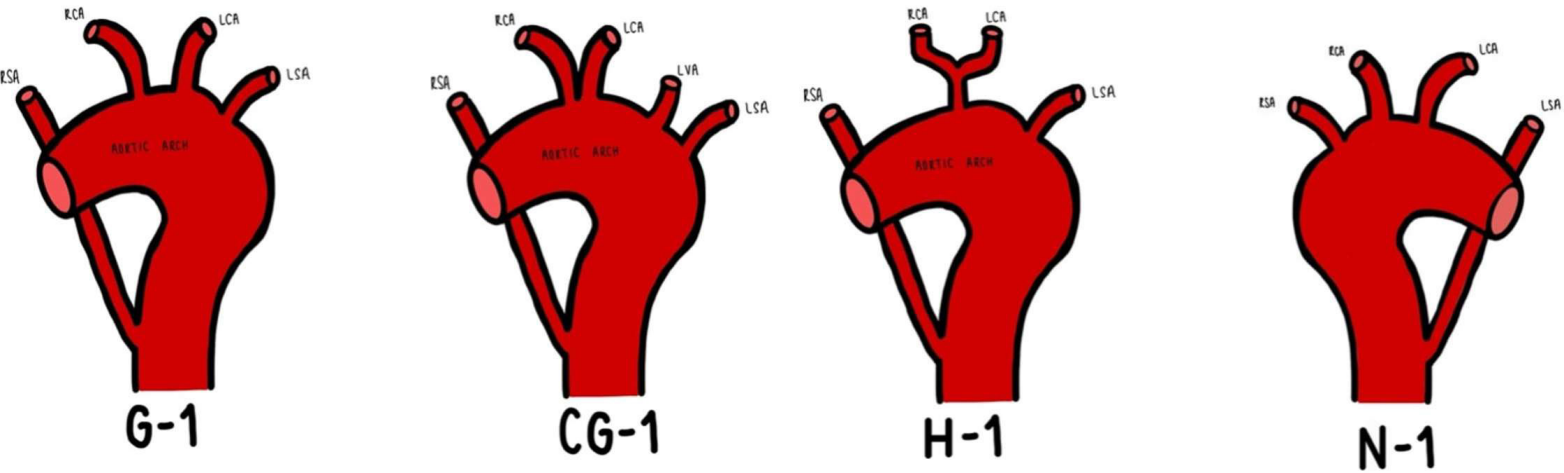
A schematic diagram of the Adachi-Williams’ Classification of aberrance of the right subclavian artery. RSA: right subclavian artery. RCA: right common carotid artery. LCA: left common carotid artery. LSA: left subclavian artery. LVA: left vertebral artery.

Aberrance of the right subclavian artery (ARSA), classically referred to as arteria lusoria, occurs in 0.4–2% of individuals, with further anatomical morphological variations present within the same classification system.^2^ According to the Adachi-Williams’ Classification (Figure 4), approximately 80% of individuals have three branches arising from the aortic arch, described as Type G: brachiocephalic trunk, left common carotid artery and left subclavian artery.^2^ The most likely variant in the present case is Type H (Figure 4), which occurs in 11% of individuals, characterised by both the left and right common carotid arteries arising from a bicarotid trunk and the right subclavian artery arising directly from the aortic arch (Figure 3).^2,3^

Like in any individual with neck injury, developing a sound clinical management plan alongside multidisciplinary team involvement was vital to optimise the care for this patient. Traditionally, a “zonal” approach to penetrating neck trauma in which neck exploration is advocated for; however, recent studies suggest that this is inefficient as the majority of surgical interventions yielded no major injuries.^4^ As a result, there has been a recent proposition of a “no zone” approach, wherein haemodynamically stable patients with penetrating neck trauma are preferred to be evaluated with CTA alongside standard clinical examination to help guide further management.^4^ The “no zone” approach has resulted in favourable outcomes and helped clinicians formulate a plan on how to proceed after confirming on radiological imaging in which structures were involved.^4^ Research suggests multidetector contrast-enhanced computed tomographic angiography (MDCT-A) to be the most effective in detecting foreign bodies; however, there is a thought to decrease success in visualising wooden objects.^5^ In the present case, the prompt decision to use CTA ensured adequate visualisation of the foreign body, since its course in the patient’s neck was clearly seen. The wood breached the platysma and entered the thorax abutting all crucial structures but did not cause any extravasation or significant damage due to the patient fortuitously having arteria lusoria. The 3D reconstruction depicts a clearer picture of its positioning amongst the surrounding vascular structures, illustrating the favourable outcome for the patient.

## Learning points

1. Penetrating neck injuries, particularly with a retained foreign body, although a rare presentation, is one that requires rapid decision-making and intervention.
2. While often in suspected arterial injury a “red-blanket” or straight to theatre transfer is warranted, careful consideration of the patient status and appropriate imaging can allow for a safer planned operative intervention.
3. A coordinated multidisciplinary approach is vital to the management of acute trauma patients to ensure that the optimum outcome can be achieved.
4. Anatomical variations, while uncommon, can correlate with significant clinical outcomes - in this case positively.
